# Experimental Approach Reveals the Role of *alx1* in the Evolution of the Echinoderm Larval Skeleton

**DOI:** 10.1371/journal.pone.0149067

**Published:** 2016-02-11

**Authors:** Hiroyuki Koga, Haruka Fujitani, Yoshiaki Morino, Norio Miyamoto, Jun Tsuchimoto, Tomoko F. Shibata, Masafumi Nozawa, Shuji Shigenobu, Atsushi Ogura, Kazunori Tachibana, Masato Kiyomoto, Shonan Amemiya, Hiroshi Wada

**Affiliations:** 1 Graduate School of Life and Environmental Sciences, University of Tsukuba, Tsukuba, Japan; 2 Institute of Biogeosciences, Japan Agency for Marine-Earth Science and Technology, Yokosuka, Japan; 3 Division of Life Science, Graduate School of Natural Science and Technology, Kanazawa University, Kanazawa, Japan; 4 Institute for Molecular Science of Medicine, Aichi Medical University, Nagakute, Japan; 5 National Institute for Basic Biology, Okazaki, Japan; 6 Center for Information Biology, National Institute of Genetics, Mishima, Japan; 7 Department of Genetics, The Graduate University for Advanced Studies, Mishima, Japan; 8 School of Life Science, The Graduate University for Advanced Studies, Okazaki, Japan; 9 Nagahama Institute of Bio-Science and Technology, Nagahama, Japan; 10 Graduate School of Bioscience, Tokyo Institute of Technology, Yokohama, Japan; 11 Marine and Coastal Research Center, Ochanomizu University, Tateyama, Japan; 12 Department of Integrated Biosciences, Graduate School of Frontier Sciences, The University of Tokyo, Kashiwa, Japan; 13 Research and Education Center of Natural Sciences, Keio University, Yokohama, Japan; Laboratoire de Biologie du Développement de Villefranche-sur-Mer, FRANCE

## Abstract

Over the course of evolution, the acquisition of novel structures has ultimately led to wide variation in morphology among extant multicellular organisms. Thus, the origins of genetic systems for new morphological structures are a subject of great interest in evolutionary biology. The larval skeleton is a novel structure acquired in some echinoderm lineages via the activation of the adult skeletogenic machinery. Previously, VEGF signaling was suggested to have played an important role in the acquisition of the larval skeleton. In the present study, we compared expression patterns of *Alx* genes among echinoderm classes to further explore the factors involved in the acquisition of a larval skeleton. We found that the *alx1* gene, originally described as crucial for sea urchin skeletogenesis, may have also played an essential role in the evolution of the larval skeleton. Unlike those echinoderms that have a larval skeleton, we found that *alx1* of starfish was barely expressed in early larvae that have no skeleton. When *alx1* overexpression was induced via injection of *alx1* mRNA into starfish eggs, the expression patterns of certain genes, including those possibly involved in skeletogenesis, were altered. This suggested that a portion of the skeletogenic program was induced solely by *alx1*. However, we observed no obvious external phenotype or skeleton. We concluded that *alx1* was necessary but not sufficient for the acquisition of the larval skeleton, which, in fact, requires several genetic events. Based on these results, we discuss how the larval expression of *alx1* contributed to the acquisition of the larval skeleton in the putative ancestral lineage of echinoderms.

## Introduction

The current diversity in animal morphology has resulted from the gradual acquisition of novel structures and, occasionally, the loss of existing structures. Despite recent advances in evolutionary developmental biology, the mechanisms by which animals acquire novel structures remain poorly understood. Herein, using echinoderm larvae as a model, we seek to explain how a novel morphological trait emerges. Echinoderm larvae provide a good opportunity to address the underlying mechanisms of morphological evolution, because they show a variety of forms between classes and their developmental mechanisms have been well-studied to date [1–3]. In this study, we focus on the acquisition of a larval skeleton in some echinoderm taxa and explore the molecular mechanism by which this novel trait has evolved.

Echinoderms include five extant classes: echinoids (sea urchins), holothuroids (sea cucumbers), ophiuroids (brittle stars), asteroids (starfish), and crinoids (sea lilies). The larvae of some groups possess skeletons that are specific to the planktonic stage. In the pluteus larvae of sea urchins and brittle stars, the remarkable larval skeletons support their long arms. However, crinoids, starfish, and sea cucumbers have bipinnaria-auricularia-type larvae [4], which lack larval arms supported by a skeleton [5, 6]. Although sea cucumber larvae have small spicules, a larval skeleton is generally accepted to be a derived structure, and the common ancestors of echinoderms are thought not to have possessed such a skeleton; the ancestral condition is retained by crinoids and (possibly) starfish [7]. This idea is consistent with the absence of a larval skeleton in hemichordate acorn worms, the sister group to the echinoderms [8–10].

At early larval stages, calcareous endoskeletons are observed in only some of the echinoderm classes, but all echinoderms have adult skeletons. Indeed, as the most basal fossil group of echinoderms has an adult endoskeleton [11, 12], such a skeleton is the most widely shared feature of the Echinodermata. Previous studies have shown that larval and adult skeletogenic cells of sea urchins express similar genes, including those encoding certain transcription factors and matrix proteins[13–17]. Thus, it has been proposed that the construction of the larval skeletons of echinoderms requires the activation of adult skeletogenic machinery in larval mesoderm cells [1, 18–20].

The development of the larval skeleton has been well studied in sea urchins (reviewed in ref. [1]). Briefly, skeletal structures are formed in primary mesenchyme cells (PMCs), which are the descendants of large micromeres. Such cells express various transcription factors (TFs), including Ets1/2, Alx1, and Tbr, which together form a skeletogenic gene regulatory network (GRN) that controls the expression levels of downstream genes involved in mesenchyme differentiation and biomineralization. However, even in starfish larvae lacking larval skeletons, a similar repertoire of TF genes is active in mesodermal cells [21–24]. These observations suggest that the acquisition of a larval skeleton involved the activation of only a few skeletogenic genes. One such candidate is the VEGF gene, which is required for larval skeletogenesis in the sea urchin [25, 26] but is not expressed in the larvae of starfish [27]. Both the VEGF receptor and the ligand thereof are expressed in brittle star larval skeletal cells and adjacent epidermal cells [27].

In the present study, we analyzed the *alx* gene encoding the (PRD class) homeobox transcription factor. In sea urchin larvae, Alx1 has been reported to be indispensable for larval skeletogenesis; the protein regulates the expression levels of many skeletogenic genes [28–30]. Our results suggest that *alx1* activation in the larval stage was also important for the evolution of the larval skeleton. Furthermore, we experimentally induced the expression of *alx1* in normally skeleton-less starfish larvae. Based on our observations, we discuss how novel and complex morphological structures evolve.

## Results

### Characterization of Echinoderm *alx* Genes

**Fig 1:**
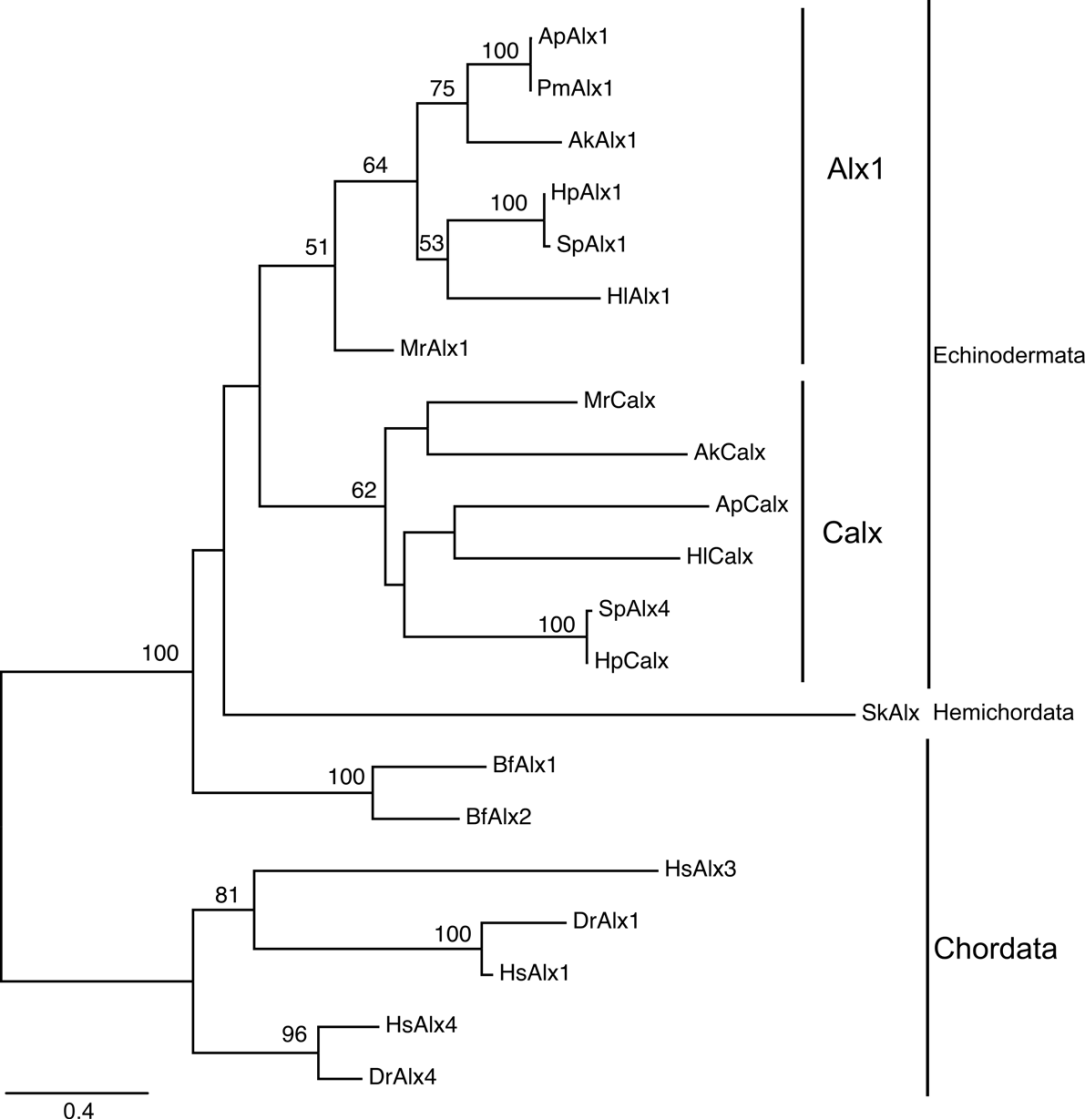
Maximum likelihood (ML) tree of Alx proteins. The tree was constructed using conserved amino acid sequences. The numbers on the nodes indicate the bootstrap values (1,000 pseudoreplicates). Values greater than 50% are shown. Ak: *Amphipholis kochii* (brittle star); Ap: *Asterina pectinifera* (starfish); Bf: *Branchiostoma floridae* (amphioxus); Dr: *Dario rerio* (zebrafish); Hl: *Holothuria leucospilota* (sea cucumber); Hp: *Hemicentrotus pulcherrimus* (sea urchin); Hs *Homo sapiens* (Human); Mr: *Metacrinus rotundus* (sea lily); Pm: *Patiria (Asterina) miniata* (starfish); Sk: *Saccoglossus kowalevskii* (acorn worm); Sp: *Strongylocentrotus purpuratus* (sea urchin).

Two adjacent *alx* genes have been identified in the sea urchin *Strongylocentrotus purpuratus* genome [31], suggesting that the genes were derived via tandem duplication. As vertebrates have at least three *alx* genes [32], we explored the orthology of the *alx* homologs of deuterostomes. Transcriptome searches revealed two *alx* paralogs in every echinoderm species examined and a single *alx* in acorn worms. Phylogenetic analysis of deuterostome Alx sequences suggested that gene duplication has occurred independently in echinoderms and vertebrates (Fig 1). Although one sea urchin paralog was annotated *alx4* because of its similarity to the vertebrate *alx4*, the evolutionary tree did not support a between-gene orthology. Thus, to avoid confusion, we designate one of the echinoderm paralogs *Calx* (Coelomic *alx*), reflecting the expression pattern thereof, as discussed below.

**Fig 2:**
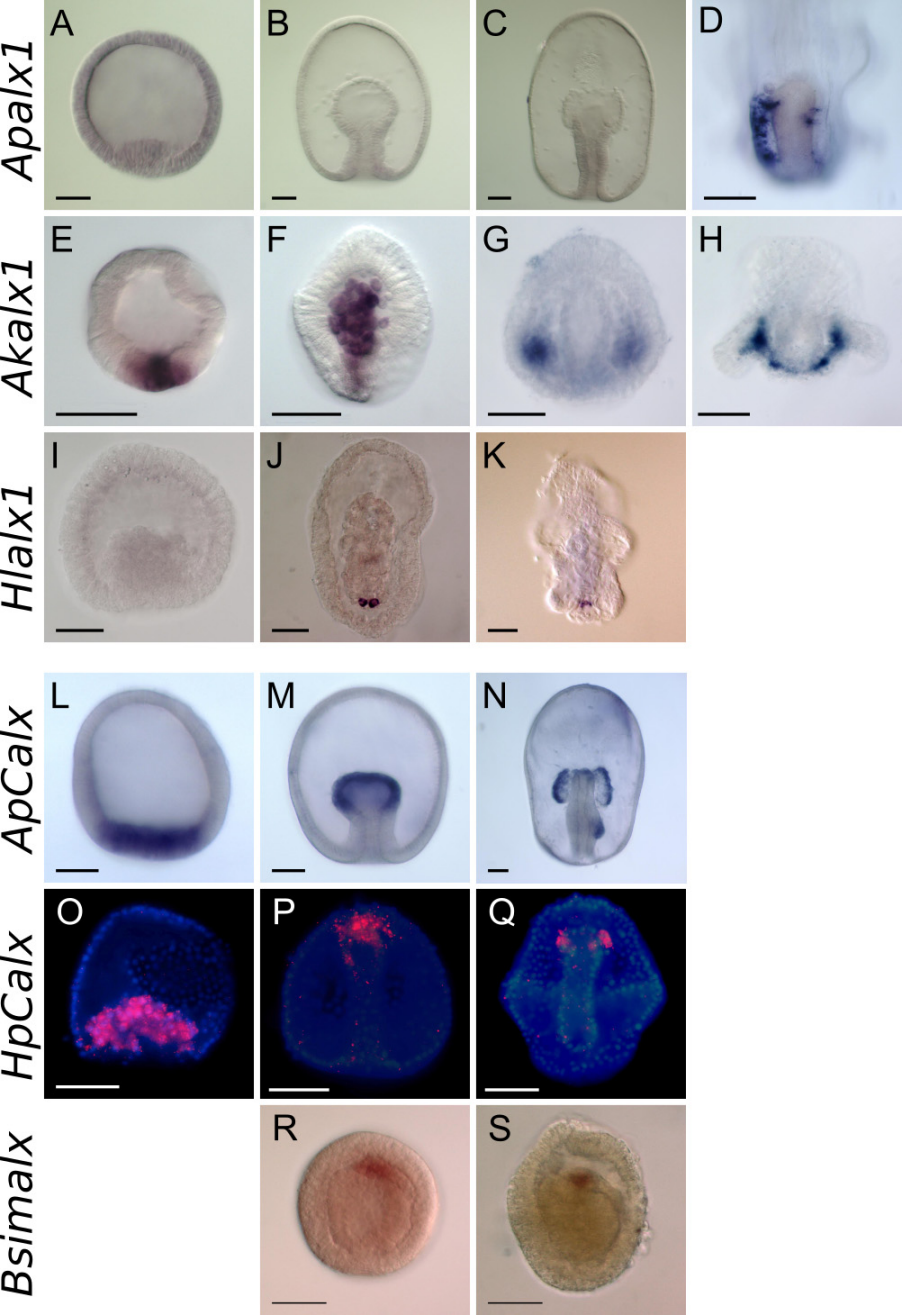
The expression patterns of *alx* genes in ambulacraria. (A–D) The expression pattern of starfish *alx1* (*Apalx1*). (A–C) Expression was barely evident prior to the bipinnaria stage. (D) Expression was evident in mesenchyme cells of the posterior region of late bipinnarial larvae. (E-H) The expression pattern of brittle star *alx1* (*Akalx1*). (E) Expression was evident in the putative vegetal plate of the blastula (F) and in mesenchyme cells that ingressed from the vegetal plate. (G) At the gastrula stage, expression was evident in mesenchyme cells located bilaterally to the archenteron. (H) In plutei, expression was evident in mesenchyme cells located along the arms. (I-K) The expression pattern of sea cucumber *alx1* (*Hlalx1*). (I) Expression was not evident prior to the gastrula stage. (J) In mesenchyme of the gastrula, expression was evident in a few cells located in the posterior larval region. (K) In auricularia larvae, expression was evident in mesenchyme cells. (L-N) The expression pattern of starfish *Calx* (*ApCalx*). (L) Expression was evident in the vegetal plate of the blastula. (M) Expression was noted at the tip of the archenteron of the gastrula. (N) In early bipinnariae, expression persisted in the coelom and de novo expression was evident in the posterior enterocoel that invaginated from the left side of the gut. (O-Q) The expression pattern of sea urchin *Calx* (*HpCalx*). Red fluorescent signals indicate *HpCalx* transcripts and blue signals denote nuclei stained by DAPI. (O) Expression was evident in ingressed primary mesenchyme cells (PMCs) of the blastula mesenchyme. (P) In late-stage gastrulae, signals disappeared from PMCs but were observed in secondary mesenchyme cells (SMCs) located around the tip of the archenteron. (Q) Expression was restricted to the coelomic pouches of prism larvae. (R, S) The expression pattern of acorn worm *alx* (*Bsimalx*). (R) In the mid-gastrula, expression was evident in cells at developing protocoel. (S) At a later stage of gastrula, expression was observed in the proximal part of the protocoel. Scale bars: 50 μm.

Previous studies showed that *alx1* is expressed in the larval skeletons of sea urchins and sea cucumbers [28, 33] and in adult sea urchin skeletogenic cells [13]. In this study, we found that *alx1* was expressed in the larval skeletogenic sites of brittle stars *Amphipholis kochii* (Fig 2E–2H) and in a different species of the sea cucumber *Holothuria leucospilota* (Fig 2I–2K), supporting the idea that Alx1 plays an essential role in echinoderm larval skeletogenesis. During development of the starfish *Asterina pectinifera*, *alx1* was first expressed at the late bipinnaria stage, when adult skeletogenesis commences (Fig 2A–2D). Alx1 was initially expressed in small spots located along the left side of the stomach between the somatocoel and the epidermis (S1 Fig). Later, expression was evident in mesenchyme cells lying along the right side of the stomach (Fig 2D). In late-stage brachiolaria, signals were evident from mesenchyme cells within the adult rudiment and exhibited a pentaradial pattern nearly identical to that of starfish *Carbonic Anhydrase1* (*ApCA1*) [19, 27] (S1 Fig). Quantitative real-time polymerase chain reaction (real-time PCR) analysis confirmed these observations: transcript levels were low before the bipinnaria stage, but they rose thereafter as development proceeded toward metamorphosis (S1 Fig). These data support the notion that Alx1 performs important roles during both adult and larval skeletogenesis. Notably, the barely detectable Alx1 expression during early starfish embryogenesis was followed by notable upregulation thereof, suggesting that both VEGF signaling and *alx1* expression during early embryogenesis are important in terms of larval skeleton development.

In contrast, starfish *Calx* was expressed in the vegetal plate, including presumptive mesoderm cells, prior to gastrulation. Expression was then evident in the tips of archenterons of the gastrulae and then in coelomic pouches until the late bipinnarial stage was attained (Fig 2L–2N). Differences in the expression profiles of *alx* paralogs were also evident in quantitative analysis; *Calx* was highly expressed from the early embryonic stage (S1 Fig). In the sea urchin *Hemicentrotus pulcherrimus*, expression of *alx4* (the *Calx* ortholog) was evident in both coelomic and skeletogenic cells at an earlier stage (Fig 2O–2Q), as previously reported in *S*. *purpuratus* [29]. This expression pattern suggests that, in echinoderms, *Calx* plays a common role in coelomic development. This idea is supported by the fact that the single *alx* gene of the acorn worm *Balanoglossus simodensis* was expressed in the coelom of mid- and late-gastrula (Fig 2R and 2S).

### Forced expression of *alx1* in starfish embryo

In the sea urchin, removal of the PMCs at the mesenchyme blastula stage causes secondary mesenchyme cells (SMCs), which usually give rise to nonskeletal mesodermal cells, to differentiate into skeletogenic cells [34]. During this process, *alx1* is expressed in transfating SMCs originally lacking expression [35, 36]. As the starfish also has mesenchyme cells that ingress from the tip of the archenteron (as do sea urchin SMCs), it is possible that the experimental induction of *alx1* expression in starfish larvae induces developmental or genetic changes associated with larval skeletogenesis. Thus, we next tested the effect of artificial expression of *alx1* in starfish larvae.

To express *alx1* in starfish larvae, we introduced mRNA encoding full-length sea urchin Alx1 (HpAlx1) into starfish eggs via microinjection. We confirmed that the injection of this mRNA into sea urchins increased skeletogenic cell numbers, as previously reported (S2 Fig) [36]. In addition, we observed that injected green fluorescent protein (GFP)-fused *Hpalx1* mRNA was successfully translated in the nuclei of starfish embryos (S2 Fig). We found that injection of large quantities of mRNA for *Hpalx1* (in solutions of over 1.5 mg/ml) often prevented gastrulation, followed by the delay in or arrest of development (S3 Fig), as observed when comparable amounts of mRNA were injected into sea urchin eggs [36, 37]. However, notably, in the sea urchin, the injection of lower levels of mRNA triggered excessive production of skeletogenic cells (S2 Fig) [36], but starfish embryos apparently did not respond at all to such low levels of mRNA (S3 Fig). We observed no phenotypic changes, and no spicules formed, when *Hpalx1*-encoding mRNA solutions at concentrations lower than 1.5 mg/ml were injected (S3 Fig), indicating that larval expression of *alx1* was not sufficient to fully activate the skeletogenic machinery.

Next, we examined whether the lack of an external phenotype was attributable to the buffering of *alx1* overexpression by the GRN or whether *alx1* overexpression did, in fact, alter gene expression. As the genetic basis of starfish development still remains to be resolved, we comprehensively analyzed alterations in gene expression using the RNAseq mode of a next-generation sequencer. RNAs extracted from 24-h-old starfish embryos (S3 Fig) into which sea urchin *alx1* mRNA (0.5 mg/ml) had been introduced were sequenced; embryos injected with GFP mRNA served as controls. To minimize potential problems associated with high-level genetic heterogeneity, we used embryos from the same parents in three experimental replications. As the genomic sequence of the species is not yet available, we first performed de novo transcriptome assembly using the whole sequence. The assembly includes 107,286 putative genes (trinity sub-components) and 206,700 transcripts (S1 Table). According to a BLASTX search with an e-value lower than 1E-5, we found that 18,282 putative genes showed similarity to sea urchin coding genes (S1 Dataset).

To quantify the levels of each transcript, we obtained read counts by mapping the reads onto the transcriptome. We confirmed that the expression level (fragments per kilo base per million reads, FPKM value) of injected *Hpalx1* mRNA was comparable to some endomesoderm TF genes, *Apets1/2*, *Apfoxn2/3*, and *Aphhex*, which were previously reported to be functional in the starfish gastrula (S4 Fig) [22, 23]. We also confirmed that *ApCalx* showed expression to some extent in this stage, although the FPKM value was low compared with other mesodermal TFs. On the other hand, endogenous *Apalx1* was fragmentally identified as three putative genes via BLASTN using the cloned sequence with the low FPKM values (S4 Fig).

**Fig 3:**
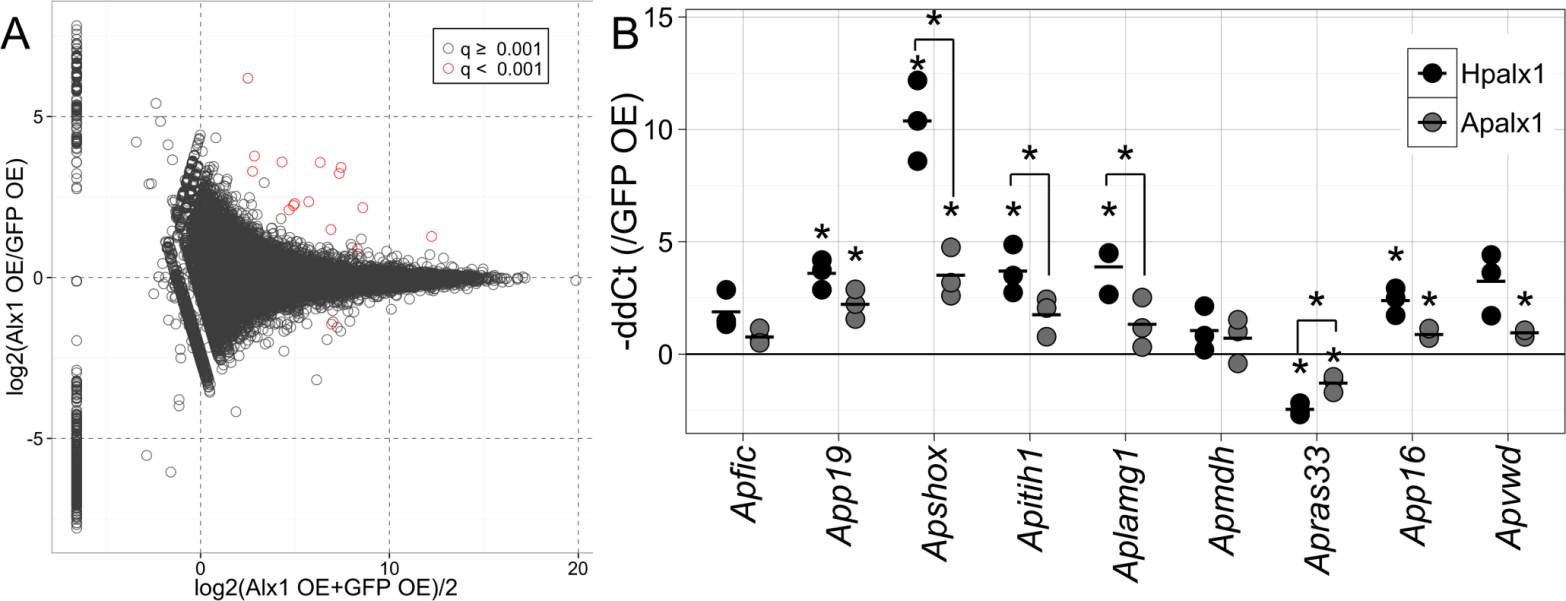
Differentially expressed (DE) genes after the injection of *alx1* mRNA into starfish. (A) MAplot of DE genes identified by RNAseq. Data represent individual putative gene in a log2 ratio versus log2 average plot. Red plots indicate DE genes with q value (FDR) < 0.001. (B) Relative expression levels in 24-h-old embryos were calculated via real-time PCR. The data show–ΔΔCt values relative to the embryos in which EGFP mRNA was overexpressed. The expression levels were normalized to that of *ApEF1a*. Data from three trials using different batches of eggs are shown. Black points indicate experimental data derived using sea urchin *Hpalx1*, and gray points show data derived using starfish *Apalx1*. The horizontal bars indicate mean values. Asterisks indicate significant differences between ΔCt values of sample pairs (paired t-test with Bonferroni correction, p < 0.05).

**Fig 4:**
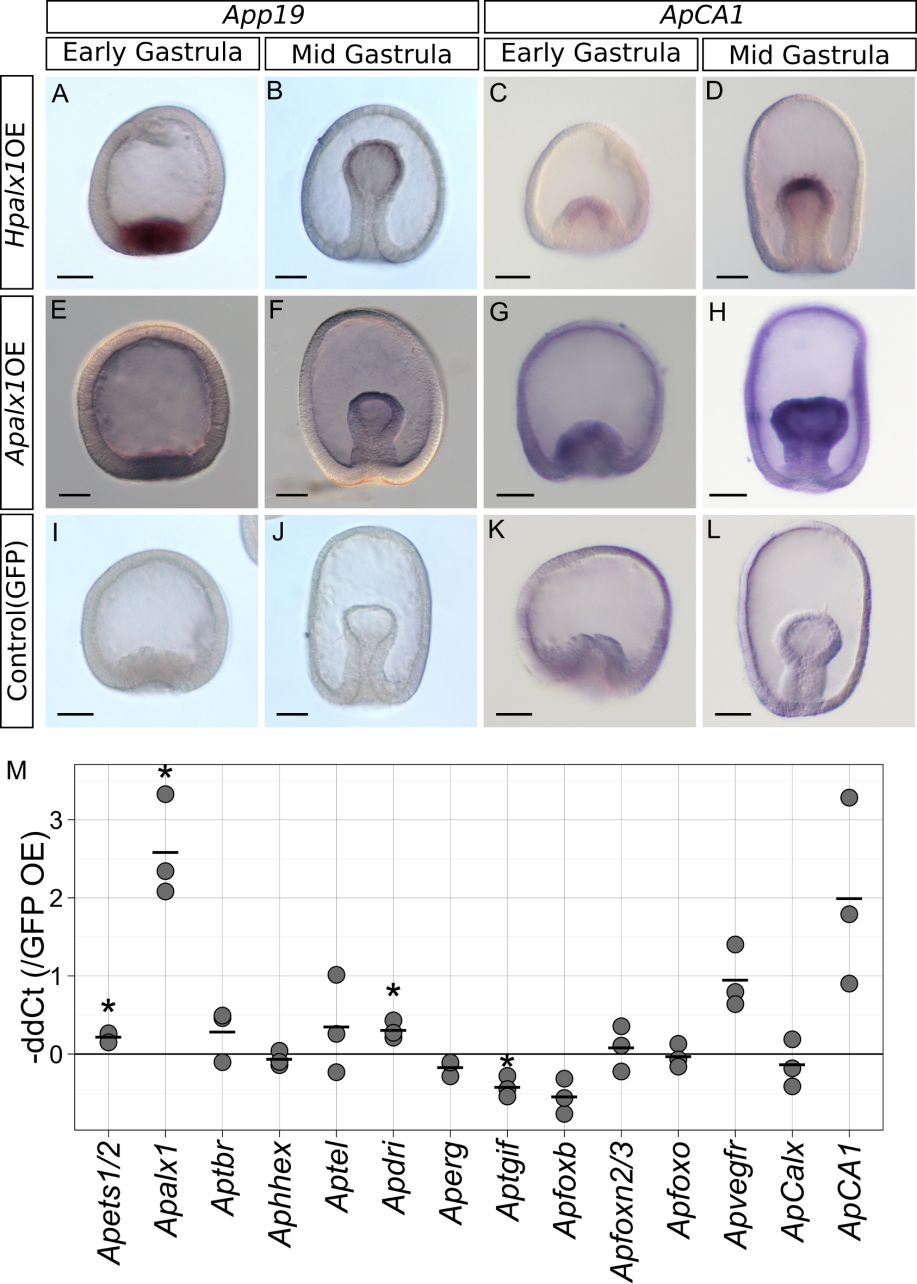
Expression patterns of putative skeletogenic genes in embryos expressing *alx1*. Solutions 0.5 mg/ml in mRNA for *Hpalx1* (A-D), 1 mg/ml in mRNA for *Apalx1* (E-G) or EGFP (I-L) were injected into starfish eggs, which were next reared for 15 h (to the early gastrula stage) or 24 h (to the mid-gastrula stage). (A) *App19* was detected in the presumptive mesoderm. (B) *App19* expression was retained by mesoderm cells at the tip of the archenteron. (C) *ApCA1* mRNA was also detected in the presumptive mesoderm. (D) Ap*CA1* expression was retained in the mesoderm of gastrulae. (E-H) The similar expression patterns to the sea urchin *Hpalx1* overexpression were observed in the starfish *Apalx1* overexpressed embryos. (I-L) Neither *App19* nor *ApCA1* was detectable in control embryos. Scale bars: 50μm. (M) Changes in the expression levels of skeletogenic gene orthologs upon injection of *Hpalx1* mRNA into starfish. Relative expression levels in the 24-h-old embryos were measured via real-time PCR. The data show -ΔΔCt values relative to those of (control) EGFP-expressing embryos. The expression level of each gene was normalized to that of the *ApEF1a* gene. Three distinct trials with different batches of eggs were run. Asterisks indicate significant difference between ΔCt values of control and *Hpalx1* overexpression (paired Student’s t-test, p < 0.05). Endogenous *Apalx1* was highly upregulated upon ectopic *Hpalx1* expression.

We identified putative differentially expressed (DE) genes by comparing the normalized read counts between samples expressing *Hpalx1* and controls (Fig 3A). A total of 16 putative genes exhibited significant transcriptional differences (false discovery rate, FDR < 1E-3; Table 1). Of these, 13 were successfully annotated because they were similar to genes in the sea urchin protein database (SpBase) or GenBank (e-value < 1E-5). To confirm the results of DE analysis by RNAseq, we used real-time PCR to measure changes in the expression levels of nine DE genes and orthologs of sea urchin skeletogenic TFs in 24-h-old embryos from three different parental pairs (Fig 3B). All DE genes exhibited alterations in expression levels consistent with the RNAseq result levels upon *Hpalx1* overexpression, although the changes were not statistically significant in some genes by real-time PCR. To determine whether DE genes were activated in the mesoderm, we also examined spatial expression patterns for some DE genes by *in situ* hybridization. However, for most genes, we found that upregulation of transcription might be not sufficient for detection by *in situ* hybridization. *App19* was the only gene whose expression was detected in *alx1*-overexpressed embryos by *in situ* hybridization. *App19* encodes a protein similar to sea urchin P19. In sea urchin, P19 is thought to be associated with biomineralization, because it is specifically expressed in PMCs in the larval stage [38–40], and also detected in adult test plates and teeth [41]. The upregulation of *App19* was observed in presumptive mesoderm cells in embryos to which sea urchin *Hpalx1* mRNA had been introduced (Fig 4A and 4B), although the injection of *alx1* mRNA induced ubiquitous translation of Alx1 protein (S2 Fig). Thus, at least for *App19*, the upregulation occurred with support from TF(s) expressed in mesoderm cells.

**Table 1:** DE genes in Alx1 over-expressed embryos.

Rank	Trinity Gene ID	mean Normalized count (± S.D.)		FDR	best hit SPU or Genbank ID	common name of the best hit gene	blastx evalue	Annotation
		Alx1 OE	EGFP OE					
1	comp134774_c0	497.88 (± 84.85)	52.93 (± 6.09)	0	SPU_017963	Sp-Tnr	0	*Apfic*
2	comp144351_c0	566.44 (± 331.31)	52.89 (± 7.25)	2.7E-25	SPU_004136	Sp-P19	3.0E-17	*App19*
3	comp142398_c0	278.73 (± 170.25)	23.36 (± 4.17)	1.6E-21	XP_005090524	uncharactarized protein	2.00E-13	*Ap142398*
4	comp135208_c0	48.45 (± 26.04)	0.66 (± 0.6)	5.2E-18	SPU_019268	Sp-Shox	2.0E-34	*Apshox*
5	comp136962_c0	119.84 (± 28.95)	23.42 (± 11.24)	4.7E-14	-	-	-	(unknown1)
6	comp140359_c0	7493.33 (± 602.34)	3096.43 (± 652.69)	9.8E-12	XP_00338253	coactsin-like	4.00E-43	*ApcactL*
7	comp136548_c2	65.7 (± 13.76)	13.95 (± 4.97)	7.8E-11	SPU_019450	Sp-Itih_2	0	*Apitih1*
8	comp136548_c1	69.85 (± 20.77)	14.23 (± 4.49)	9.6E-11	SPU_027343	Sp-Itih_6	2.0E-39	*Apitih2*
9	comp140934_c0	68.87 (± 52.87)	5.73 (± 0.73)	7.1E-10	SPU_015404	Sp-LamG/Egff3	0	*Aplamg1*
10	comp142546_c0	817.07 (± 193)	181.64 (± 115.8)	7.3E-10	XP_001636622	predicted protein	0	*Apmdh*
11	comp136548_c0	53.25 (± 7.87)	12.44 (± 4.35)	1.4E-08	SPU_027343	Sp-Itih_6	0	*Apitih3*
12	comp136524_c0	200.43 (± 54.96)	71.4 (± 17.46)	1.4E-07	SPU_021744	Sp-Vwd/Fa58c	1.0E-19	*Apvwd*
13	comp132292_c0	26.73 (± 18.43)	1.95 (± 1.73)	2.2E-07	-	-	-	(unknown2)
14	comp151049_c0	73.86 (± 27.57)	201.82 (± 22.57)	2.2E-07	SPU_006392	Sp-Rab33	0	*Aprab33*
15	comp125517_c0	21.08 (± 13.65)	2.14 (± 1.19)	4.5E-05	SPU_018408	Sp-P16	6.00E-11	*App16*
16	comp141693_c0	428.81 (± 44.26)	232.06 (± 36.71)	5.8E-04	-	-	-	(unknown3)

Next, we examined whether induced expression of *alx1* altered the expression of any orthologs of sea urchin skeletogenic GRN. In sea urchins, two TF genes, *dri* and *foxb*, were shown to be positively regulated by Alx1 [30, 42]. The ortholog of *dri* was slightly upregulated in *Hpalx1*-overexpressed starfish embryos, whereas the expression of *foxb* was not significantly affected (Fig 4M). In the normal development of starfish gastrulae, *Apdri* is expressed in mesoderm and oral ectoderm, and we observed a specific upregulation in the mesoderm cells of the *Hpalx1*-overexpressed early gastrulae (S5 Fig). Among skeletogenic TFs, endogenous starfish *Apalx1* was the only ortholog of skeletogenic TF gene that was strongly upregulated by exogenous sea urchin *Hpalx1* (Fig 4M); this effect was unexpected because negative autoregulation was previously evident in the sea urchin embryo [28–30, 42]. *Apvegfr*, a VEGF receptor gene, was also slightly upregulated upon *Hpalx1* overexpression. This is consistent with the sea urchin GRN, where *vegfr* is positively regulated by Alx1 [29, 30]. However, in starfish, the upregulation was not significant. We identified the carbonic anhydrase gene (*ApCA1*) as a useful marker of starfish adult skeletogenesis [19, 27]. *ApCA1* expression was upregulated in presumptive mesodermal cells when mRNA for *Hpalx1* was injected, as seen in *App19* (Fig 4C and 4D).

We also explored whether starfish *Apalx1* induced similar alterations in gene expression. Externally, the overexpression of starfish *alx1* showed similar effects on development: there were no obvious effects with lower (≤ 0.8mg/ml) concentrations of mRNA, and a delay in development was observed with higher (≥ 1.5 mg/ml) concentrations (S3 Fig), although complete inhibition of gastrulation was not observed in the starfish *Apalx1*-overexpressed embryos. In terms of gene expression, the injection of a 1.0-mg/ml starfish *Apalx1* mRNA solution yielded almost identical results to the injection of sea urchin *Hpalx1*. Real-time PCR showed that most DE genes were up- or downregulated in the manner noted after injection of sea urchin *alx1* (Fig 3B). However, the extent of the increases or decreases in expression was moderate in *Apshox*, *Apitih*, *Aplamg1*, and *Apras33*. The expression of *App19* and *ApCA1* was activated in mesoderm cells by starfish *Apalx1*, as by *Hpalx1* (Fig 4D–4E). Additionally, *Apdri* was upregulated specifically in the mesoderm of early gastrulae, as in the overexpression of sea urchin *Hpalx1* (S5 Fig).

### Expression patterns of DE genes

**Fig 5:**
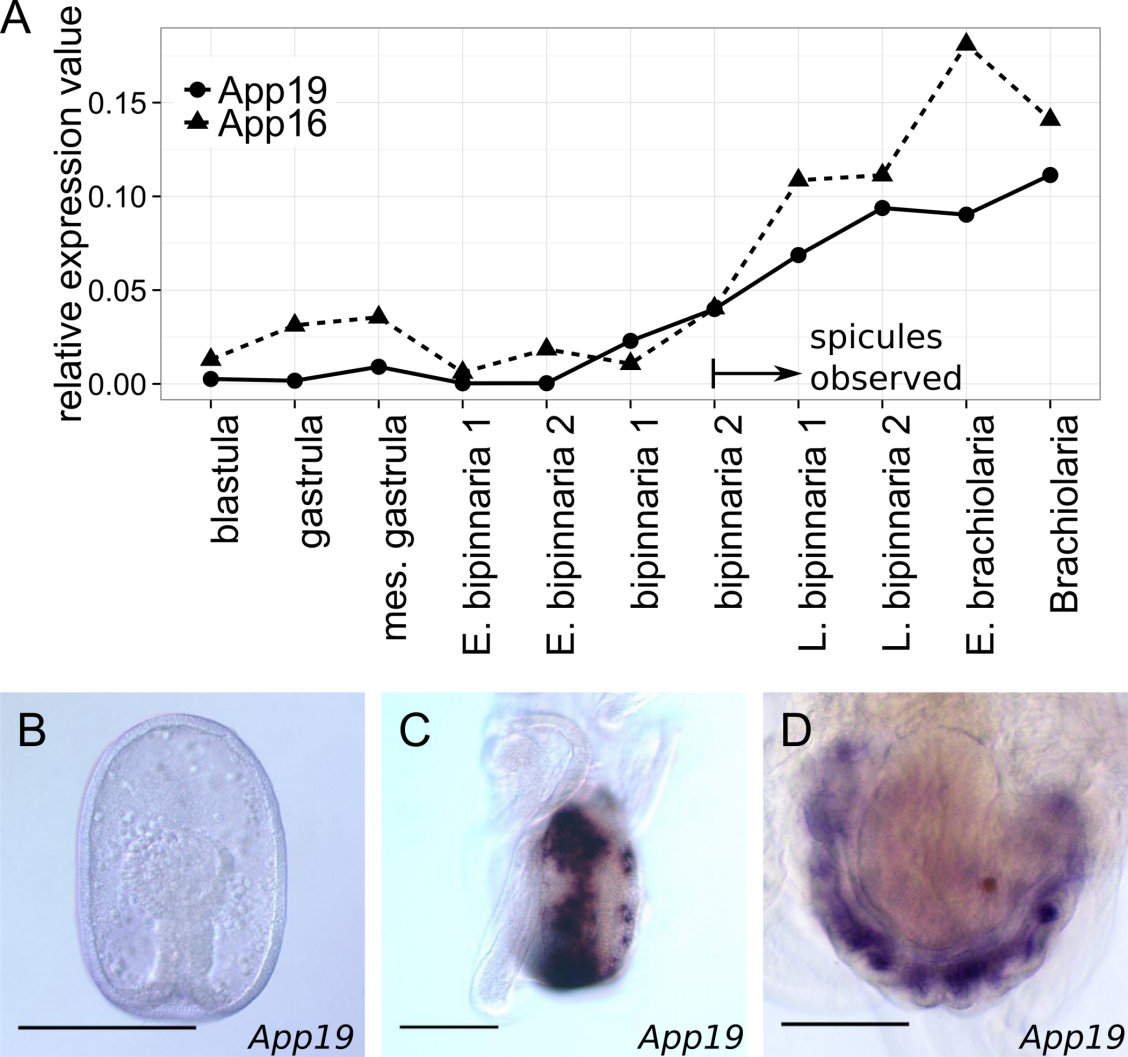
The expression patterns of *App19* and *App16*. (A) Changes in the expression of *App19* and *App16* during larval development of starfish. The expression levels shown are relative to those of *ApEF1a*. (B–D) Spatial *App19* expression patterns. (B) No expression was evident in the gastrula. (C) Signals were emitted from two rows of mesenchyme cells located in the posterior regions of the bipinnaria (an aboral view). (*E*) Signals were emitted from mesenchyme cells that were dispersed radially at the brachiolarial stage (an aboral view of the adult rudiment). Scale bars: 100μm.

Of the DE gene homologs, *p19*, *p16*, *fic*, and *cara7A* (*Carbonic Anhydrase*) have been suggested to be involved in larval skeletogenesis of the sea urchin *S*. *purpuratus* [29, 30, 38, 40, 43, 44]. In addition, the expression of an *Aplamg1* homolog, *LamG/Egff2*, was enriched in the larval skeletogenic cells of the sea urchin [30]. In starfish, *App19* expression was evident in the adult rudiment (Fig 5), as also observed for *Apalx1* (S1 Fig, ref. [13]) and *ApCA1* [27]. Although we were unable to detect *App16*, *Apfic*, or *Aplamg1* via *in situ* hybridization during the starfish metamorphosis stage, these genes were highly expressed in adult tissues containing skeletogenic cells (i.e., adult spines and adult plates, S6 Fig). No expression was detected for the remaining DE genes during metamorphosis. The expression profile of *Apmdh*, a putative metal-dependent hydrolase gene, was similar to that of *Aplamg1* and other genes in adult tissues (S6 Fig). However, the expression pattern of *Apras33* was relatively uniform. No function of *Apshox*, *Apitih1*, or *Apvwd* could be inferred from the expression patterns observed in adult tissues.

## Discussion

### Evolution of the echinoderm larval skeleton and the *alx* gene

In this study, we searched for genes involved in the acquisition of the larval skeleton by identifying genes expressed in sea urchin larval skeletogenic cells that were essential for the development of the larval skeleton but whose starfish orthologs were expressed only in adult skeletogenic cells and not at the embryonic stage. Using these criteria, previous work reported that VEGF signaling might play an important role [27]. Here, we demonstrated that activation of *alx1* also might be essential for the acquisition of the larval skeleton. Alx1 was required for the larval skeletogenesis of sea urchin [28], sea cucumber [33], and, possibly, brittle star, but it was barely expressed in starfish embryos, being instead highly expressed during adult skeletogenesis. Previously, the *alx1* gene was reported to be expressed in larval mesoderm and then coelomic pouches in a closely related species, *Patria* (*Asterina*) *miniata*, by means of *in situ* hybridization [33]. However, our quantitative data (real-time PCR and RNAseq) did not support the expression of the *alx1* gene in embryonic and early larval stage of the starfish.

Notably, we found that *alx* might have been tandemly duplicated in the echinoderm lineage. In the sister taxa of echinoderms, the hemichordates, a single *alx* gene, is expressed in the embryonic coelom. In echinoderms, the expression of *alx1* is highly specific for the skeletogenic cells of both adult and larvae (if such larval cells exist). However, starfish *alx1* is also expressed in the tube foot (S6 Fig); unlike sea urchins, the starfish lack tube foot spicules. As *Calx* is expressed in coelomic cells, a parsimonious argument is as follows: *alx* originally functioned in coelomic development, and one duplicated paralog acquired a novel function in skeletogenesis. *alx* is deeply involved in the formation of two novel structures: the larval skeleton and the calcareous skeleton *per se*.

### Partial activation of skeletogenic GRN in starfish larva by overexpression of *alx1*

We found that ectopic activation of *alx1* at early embryonic stages induced changes in the expression of a small set of genes in starfish larvae. Notably, these DE genes include several genes that are likely involved in skeletogenesis. Expression of *App19* and *ApCA1*, the homolog of sea urchin sekeletogenic genes *Spp19* and *Spcara7*, respectively, are activated in the skeletogenic cells of metamorphosing starfish larvae. In addition, these genes were specifically upregulated in embryonic mesoderm cells by the artificial and ubiquitous expression of *alx1*, suggesting that Alx1 activated these genes in cooperation with other TFs in presumptive mesoderm cells, as they did in the skeletogenic cells. Sea urchin homologs of *App16* (*Spp16*, *Spp16rel1/2*), *Apfic (Spficolin)*, and *Aplamg1* (*SpLamG/Egff*3) are also expressed in larval skeletogenic cells. Although we did not detect their expression during metamorphosis in starfish, these genes are expressed in adult skeletogenic tissues in which *alx1* is also expressed (S6 Fig). It should be noted, however, that our experiments induced expression of *alx1* in the entire embryo, and we do not have evidence to demonstrate the upregulation of these genes in mesoderm. Thus, we should be cautious in inferring that upregulation of these genes truly reflect activation of larval skeletogenesis. It is also noteworthy that the sea urchin homolog(s) of *p16*, as well as *p19* and *CA1*, are positively regulated by Alx1 in the skeletogenic GRN of sea urchin larvae [29, 43]. The expression changes of these genes by *alx1* overexpression may reflect conserved regulatory connections between them and Alx1 in starfish and sea urchin skeletogenic GRN.

### Multiple genetic steps for the acquisition of the larval skeleton

Because the larval expression of *alx1* did not trigger complete skeletogenesis, *alx1* activation may not be sufficient for acquisition of the larval skeleton. This is not surprising when considering the present architecture of the skeletogenic GRN of sea urchin larvae. A recent study revealed that VEGF signaling plays a role in skeletogenesis by regulating biomineralization in the skeletogenic cells of sea urchin embryo [25]. On the other hand, we previously reported that starfish larvae lack the expression of both VEGF ligand and receptor orthologs [27]. Thus, VEGF signaling is an obvious candidate “missing factor” that may be required for the acquisition of larval skeleton.

We consider it likely that *alx1* activation and VEGF signaling were recruited independently, because *alx1* overexpression only moderately upregulated the *vegfr* gene. Furthermore, in sea urchin, VEGF signaling is triggered by a VEGF ligand synthesized by epidermal cells [26]. Thus, *alx1* activation is not directly linked to VEGF ligand synthesis. Moreover, downregulation of VEGF signaling did not affect *alx1* expression [25]. These data imply that multiple steps (at least three steps) of gene co-option are required for the acquisition of larval skeleton.

Although the activation of several upstream regulators, including Alx1 and VEGF signaling, can account for a modular heterochronic activation of adult skeletogenic GRN, it is not certain whether these *trans* changes are sufficient for the acquisition of the larval skeleton. It is possible that some downstream effector genes, which are not involved in adult skeletogenesis, are required specifically for the larval skeleton. In this case, acquisition of cis-regulatory elements for these effectors might also be involved in the evolution of the larval skeleton. Further elucidation of the mechanisms of both adult and larval skeletogenesis among echinoderms will provide greater insight into the processes involved in the acquisition of the larval skeleton.

The phenomenon of larval skeleton acquisition provides a unique opportunity to consider the evolution of novel morphological traits. Overexpression of *alx1* in starfish embryos yielded two notable results. First, such overexpression activated several genes, including possible skeletogenic genes. Second, despite the upregulation of these genes, no phenotypic changes were apparent. Canalization or robustness of the GRN may explain the evolution of traits that require multiple genetic steps [45–47]. That is, perturbation at the level of a single TF may not affect gene expression patterns, because the complex GRN buffers the perturbation [48, 49]. Ectopic activation of a single TF is, thus, a near-neutral variation. However, our present work suggests that this is not always the case. Ectopic activation of *alx1* did in fact induce ectopic expression of several genes, including some possibly involved in skeletogenesis. However, this does not necessarily indicate that *alx1* activation is seriously disadvantageous.

Recently, notable variations among the developmental genes in natural populations have been described using the sea urchin [50, 51]. Garfield et al. revealed that a number of developmental genes, including skeletogenic genes, showed variations in larval expression profiles among natural populations of sea urchin [52]. This implies that populations of ancient echinoderm species also sustained various alleles, including those modifying the expression of a developmental gene. Although it would be naive to interpret the lack of an external phenotype as evidence for the absence of any negative adaptive value, our observations suggest that an allele that induces larval expression of *alx1* can be sustained in natural populations of an ancestral echinoderm prior to the acquisition of the larval skeleton, as gene addition is seemingly near-neutral or only slightly disadvantageous. It is also possible that larval expression of *alx1* might be adaptive if up- or downregulation of the DE genes could increase embryonic fitness in certain environments in the past, although the functions of the DE genes need to be elucidated for further discussion. In either case, the ability of natural populations to accommodate variation in gene expression patterns may drive the evolution of complex genetic traits.

## Materials and Methods

### Animal Collection and Culture

Sea urchins (*Hemicentrotus pulcherrimus*) were collected in Kominato (N 35.11, E140.20), Chiba Prefecture (authorized by the Marine and Coastal Research Center, Ochanomizu University) or were provided by the Marine and Coastal Research Center, Ochanomizu University. Mature eggs and sperm were obtained by injection of 1 mM acetylcholine into adult coelomic cavities. Eggs were fertilized and reared at 16°C in artificial seawater (ASW; Tomita Pharmaceutical).

Starfish (*Asterina pectinifera*) were collected in Tateyama (N 34.99, E 139.83), Chiba Prefecture (authorized by the Marine and Coastal Research Center, Ochanomizu University), Asamushi (N 40.90, E 140.86), Aomori Prefecture (authorized by the Research Center for Marine Biology, Asamushi, Tohoku University), and Kashima (N 35.94, E 140.69) and Hiraiso (N 36.35, E 140.61), Ibaraki Prefecture (No specific permits was required for collecting this species at this locality, as the field activity did not involve protected or endangered species). Ovaries or testes were resected from mature adults. Mature eggs were obtained by treating ovaries with 1 × 10^−6^ M 1-methyladenine in ASW. Eggs were fertilized with collected sperm and reared at 23°C in ASW. For long-term culture, a synchronous motor fitted with a propeller was used to continuously agitate the seawater. Larvae were fed diatom, *Chaetoceros calcitrans* (Marine Technology).

Brittle stars (*Amphipholis kochii*) were collected in Himi (N 36.96, E 137.05), Toyama Prefecture (No specific permits was required for collecting this species at this locality, as the field activity did not involve protected or endangered species). Mature eggs were obtained by cold (4°C) and subsequent heat (23°C) shock of mature females, as described previously [53]. Sperm was acquired by dissecting males. After fertilization, embryos were reared in ASW at 23°C.

Sea cucumbers (*Holothuria leucospilota*) were collected in Shirahama (N 33.69, E 135.34), Wakayama Prefecture (authorized by the Field Science Education and Research Center, Seto, Kyoto University). Ovaries and testes were resected from mature adults via dissection. Eggs matured upon treatment with 1 mM dithiothreitol (DTT) in ASW. Fertilized eggs were raised in ASW at 28°C.

Sea lilies (*Metacrinus rotundus*) were collected by fishnet in Tokyo bay (authorized by the Marine and Coastal Research Center, Ochanomizu University). Artificial fertilization and rearing of embryos were carried out as previously described [6].

Acorn worms *Balanoglossus simodensis* were collected in Shimoda (N34.66, E138.97), Shizuoka Prefecture (authorized by the Shimoda Marine Resaerch Center, University of Tsukuba). We induced spawning by NaOH treatment as previously described [54], then fertilized gametes artificially.

### Gene Isolation

Total RNAs of larval or adult tissues were extracted using the TRIzol reagent (Life Technologies). Reverse transcription employed Primscript (TaKaRa) and a mixture of oligo-dT and random hexamer primers. Target sequences were amplified using the primer set listed in S3 Table, and certain genes were amplified via 3′-RACE using a SMART RACE cDNA amplification kit (TaKaRa). Amplified fragments were cloned into the pGEM-T Easy Vector (Promega) or pZErO-2 (Life Technologies).

### Transcriptome Sequencing and Assembly

To gather comprehensive sequence information, we performed RNAseq on sea cucumber (*H*. *leucospilota*), and sea lily (*M*. *rotundus*). RNAs were extracted from several developmental stages of each animal using RNeasy kits (Qiagen) for sea cucumber and Trizol reagent (Life technologies) for sea lily. Paired-end libraries 200 bp in insert length were prepared, and libraries were sequenced on a Hiseq2000 platform (Illumina). Library preparation and sequencing were performed by the Beijing Genomics Institute (BGI). The short reads obtained were deposited in the DDBJ Sequence Reads Archives (DRA002579 for sea cucumber and DRA002580 for sea lily). The reads were assembled by Trinity [55] (S1 Table).

### Phylogenetic Analysis

All sequences were aligned using Probalign [56], and effective sites were selected by trimAL using a gap threshold value (–gt) of 0.7 [57]. A best-fitting amino acid substitution model and a maximum likelihood (ML) tree were inferred using RAxML 8.1.1 [58]. Confidence values were calculated after 1,000 bootstrap runs. The sequences used for the analysis are listed in S2 Table.

### *In Situ* Hybridization

Digoxigenin (DIG)-labeled antisense RNA probes were transcribed from template RNAs using an appropriate RNA polymerase (T7 polymerase, TaKaRa or SP6 polymerase, Promega) and a DIG RNA labeling mix (Roche). Embryos were fixed in 4% (w/v) PFA in MOPS buffer (0.1 M MOPS, 0.5 M NaCl, and 0.1% [v/v] Tween 20). After several washes in MOPS buffer, the solution was replaced with hybridization buffer (6× SSC, 50% [v/v] formamide, 0.1% [v/v] Tween 20, 5× Denhardt’s solution, and 100 μg/ml yeast tRNA) with 0.1–1 ng/ml of the antisense RNA probe. The probes were hybridized at 50–60°C for 1 week. After hybridization, the probes were washed off in a series of SSC buffers (4× SSC, 2× SSC, or 1× SSC with 50% [v/v] formamide and 0.1% [v/v] Tween 20) at the hybridization temperature; the embryos were next washed with MOPS buffer. To detect alkaline phosphatase (AP), samples were treated with AP-conjugated anti-DIG antibody (Roche) at 4°C overnight and washed several times in MOPS buffer. Finally, AP activity was detected upon addition of NBT/BCIP solution (Roche). A peroxidase-conjugated anti-DIG antibody (Roche) and the TSA fluorescein system (Perkin Elmer) were used to detect fluorescence.

### Microinjection

The full-length *alx1* genes of the sea urchin (*H*. *purcherrimus*: *Hpalx1*) and starfish (*A*. *pectinifera*: *Apalx1*) and the full-length EGFP gene were cloned into the pBS-RN3 vector, which carries the untranscribed region (UTR) of a *Xenopus* globin gene [59]. The primer sets used are shown in S3 Table. Each insert containing a coding region and a UTR was amplified to serve as a template for in vitro transcription. mRNAs were transcribed using an mMESSAGE mMACHINE T3 Transcription Kit (Life Technologies), purified using a MEGAclear Kit (Life Technologies), and adjusted to the desired concentrations in RNase-free water. mRNA solutions were injected into unfertilized eggs of starfish or sea urchins. As a control, mRNA encoding EGFP was injected. Injected eggs were fertilized and reared under appropriate conditions, as described above.

### Immunohistochemistry

Embryos were fixed in 4% (w/v) PFA in MOPS buffer. After washing with phosphate-buffered saline (PBS) with 0.1% (v/v) Tween 20 buffer (PBST), samples were exposed to P4 antibody in a solution containing 0.5% (w/v) of blocking reagent (Roche) and next treated with Alexa Fluor 555 goat anti-mouse IgG antibody (Life Technologies) [60]. Stained samples were washed several times with PBST and observed under a fluorescence microscope.

### Quantitative Real-Time PCR

For real-time PCR, total RNAs were extracted using an RNeasy Kit featuring on-column DNase treatment (Qiagen). RNA concentrations were adjusted to 4 ng/run. The RNAs were reverse-transcribed using a PrimeScript RT Kit (TaKaRa) and treated with RNase H (TaKaRa). Real-time PCR was performed using an ABI HT7900 system and the Power SYBR Green PCR Master Mix (Life Technologies). The expression level of each target gene was normalized by reference to the Ct value of the *EF1alfa* gene using an appropriate control primer set (EF1a_1 to EF1_4 in S4 Table), the amplification efficiency of which approximated that of the target primer set.

### Quantitative RNAseq

To obtain RNAseq samples, solutions containing 0.5 mg/ml of *Hpalx1* or *EGFP* mRNA were injected into starfish eggs. The eggs were fertilized and reared at 23°C for 24 h. Total RNAs were extracted from the larvae using an RNeasy Kit featuring on-column DNase treatment (Qiagen). Three distinct sample sets were collected from the progeny of a single pair of parents. mRNAs were purified from total RNAs by binding (twice) to Dynabeads Oligo(dT)_25_ (Life Technologies). Paired-end libraries featuring insertions 200 bp in length were prepared using a TruSeq RNA Sample Preparation Kit (Illumina). The libraries were sequenced on a Hiseq 2000 platform (Illumina). Library preparation and sequencing were performed at the National Institute of Basic Biology (NIBB) and the reads were deposited in the DDBJ Sequence Reads Archives (DRA002578)

After the elimination of low-quality reads using the NGS QC Toolkit [61] the reads were assembled into a reference transcriptome with the aid of Trinity [55]. Contaminating sequences were identified by BLASTN searches against a bacterial database and removed from the contigs. The reads from each sample were mapped and counted using RSEM [62]. Normalization and detection of differentially expressed (DE) genes were achieved using the TCC package running edgeR [63, 64]. DE genes and other contigs were annotated based on the results of BLASTX searches of the sea urchin protein database and the NCBI nonredundant protein database.

## Supporting Information

S1 Dataset Result table of DE analysis. (GZ)

S2 Dataset FPKM values of putative genes. (GZ)

S1 Fig Expression patterns of *alx* genes in starfish. (*A*) *Apalx1* expression became detectable at the bipinnaria stage in mesenchyme cells and the left somatocoel, as indicated by the arrowheads (an aboral view). (*B*) At the brachiolaria stage, high-level expression was evident in the mesenchyme cells of the adult rudiment. The expressing cells seemed to be clustered in a pentaradial manner (an aboral view of the adult rudiment). (*C*) Quantitative expression profiling of two *alx* paralogs. The data show the ratio to the expression level of the *ApEF1a* gene. No data are presented for some growth stages at which we could not detect any PCR amplification because the target transcript levels were low. Scale bars: 50μm.
(TIF)

S2 Fig Validation of the *Hpalx1* mRNA injection strategy. (*A*, *B*) Skeletogenic cells were stained by P4 antibody that recognizes Msp130 protein in 48 h postfertilization (hpf) of sea urchin larvae in which *EGFP* or *Hpalx1* was overexpressed. (*A*) When mRNA encoding EGFP was injected, the embryos developed into normal plutei, with aligned skeletogenic cells. (*B*) When mRNA encoding HpAlx1 was injected, the embryos were abnormal, in that excessive numbers of skeletogenic cells were produced. (*C*) A fusion mRNA encoding both HpAlx1 and EGFP was injected into starfish eggs (the solution was 0.5 mg/ml in mRNA). EGFP signals were observed in almost all nuclei of a 15 hpf larva upon confocal microscopy. Magenta indicates staining for phalloidin. Scale bars: 50 μm.
(TIF)

S3 Fig Phenotypes of starfish embryos into which *Hpalx1* mRNA was injected. (*A–D*) mRNAs were injected at various concentrations; 24 hpf (hours postfertilization) larvae are shown. Scale bar: 50 μm. (*A*) When a concentrated (3 mg/ml) *EGFP* mRNA solution was injected, larvae developed normally to the gastrula stage. (*B*, *C*) When a lower concentration (0.1–0.8 mg/ml) of *Hpalx1* mRNA was used, the injected larvae developed normally like the controls. (*D*) When a concentrated (2 mg/ml) *Hpalx1* mRNA solution was injected, many larvae exhibited disturbances in gastrulation. The embryos shown did not commence gastrulation. (*E*, *F*) The graphs indicate the numbers of larvae exhibiting certain phenotypes when (E) sea urchin *Hpalx1* mRNA or (F) starfish *Apalx1* mRNA solutions of various concentrations were injected. All phenotypes were assessed at 48 hpf.
(TIF)

S4 Fig RNAseq quantification of starfish genes. FPKM values calculated from read mapping results were shown (mean ± S.D.). In the *Hpalx1*-overexpressed sample, injected mRNA was detected and showed similar expression level to *ets1/2*, *hhex* and *foxn2/3*. While three *Apalx1* fragments exhibited quite low FPKM values, *ApCalx* showed higher expression level.
(TIF)

S5 Fig The expression pattern of *Apdri*. Solutions 1 mg/ml of EGFP (A, D), 0.5 mg/ml in mRNA for *Hpalx1* (B, E) or 1 mg/ml in mRNA for *Apalx1* (C, F) were injected into starfish eggs, which were next reared for 15 h (to the early gastrula stage) or 24 h (to the mid-gastrula stage). (A) The expression was observed in the invaginating archenteron and putative oral ectoderm in control early gastrula. (B) Sea urchin alx1 over expressed embryos showed basically same expression pattern as control embryo. The putative oral ectoderm is far side in this picture. (C) Starfish *alx1*-overexpressed embryos showed the same expression pattern as control. (D) In mid gastrula stage, the expression was observed in mesoderm tissue at the tip of archenteron as well as putative oral ectoderm. (E, F) Almost identical expression patterns were observed in both sea urchin and starfish alx1 overexpressed gastrulae.
(TIF)

S6 Fig The expression patterns of differentially expressed (DE) genes in adult tissues. Expression levels relative to that of the *EF1a* gene were measured via real-time PCR. Results from three individual females are shown. Four skeletal tissues were examined: the dorsal (aboral) plates (DPs), the dorsal spines (DSs), the ventral (oral) plates (VPs), and the ventral spines (VSs). We also tested four nonskeletal tissues: the liver (LV), stomach (ST), ovaries (OV), and tube feet (TF).
(TIF)

S1 Table Results of the *de nove* transcriptome assemblies. (DOCX)

S2 Table Alx proteins used in the phylogenetic analysis. (DOCX)

S3 Table PCR primers for isolation of *alx* and other genes. (DOCX)

S4 Table Primer sets for real-time PCR. (DOCX)
