# Supplementary figures and images for: Experimental Approach Reveals the Role of alx1 in the Evolution of the Echinoderm Larval Skeleton
Source: PLoS One. 2016 Feb 11;11(2):e0149067. doi: 10.1371/journal.pone.0149067 (PMC4750990; doi:10.1371/journal.pone.0149067)

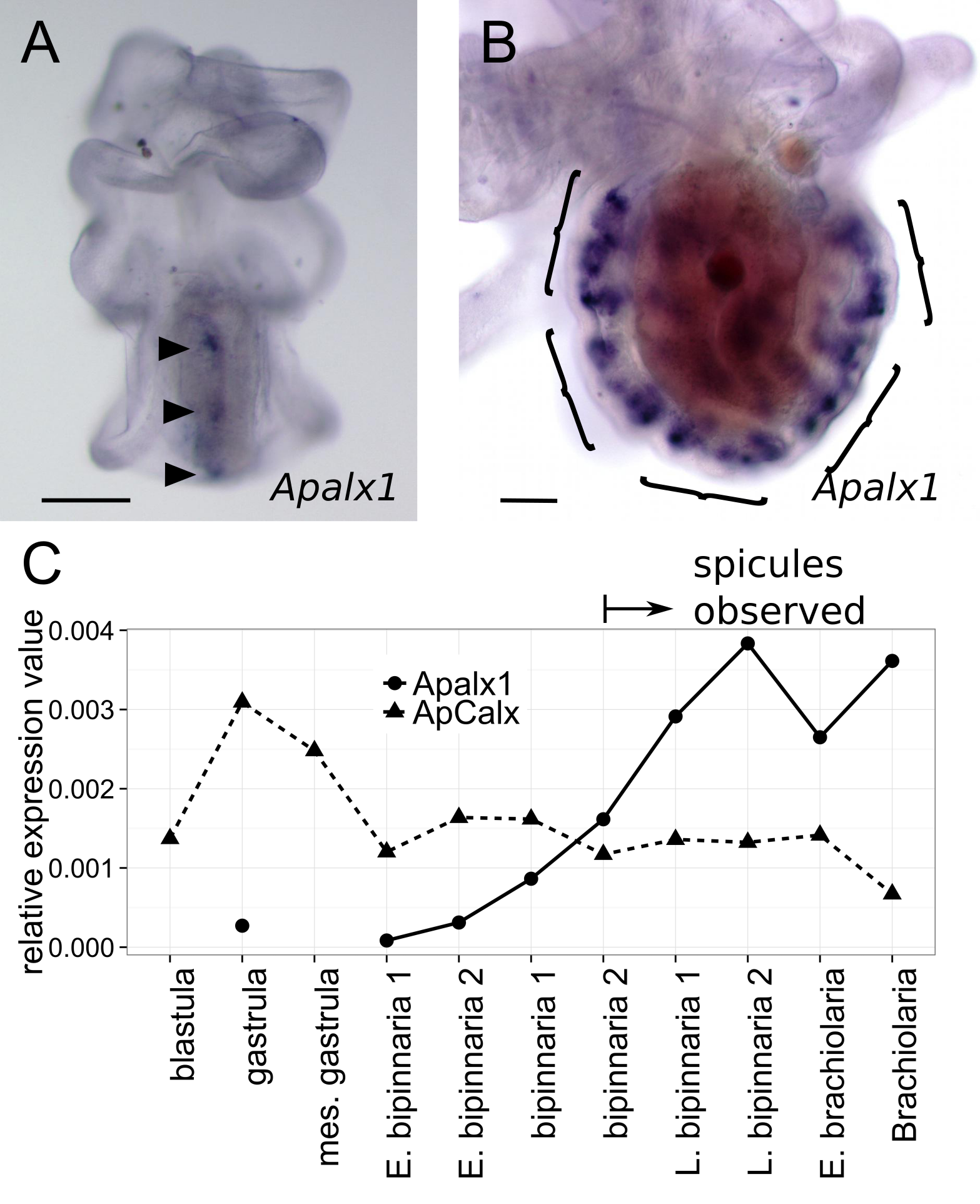

Supplement: S1 Fig — (A) Apalx1 expression became detectable at the bipinnaria stage in mesenchyme cells and the left somatocoel, as indicated by the arrowheads (an aboral view). (B) At the brachiolaria stage, high-level expression was evident in the mesenchyme cells of the adult rudiment. The expressing cells seemed to be clustered in a pentaradial manner (an aboral view of the adult rudiment). (C) Quantitative expression profiling of two alx paralogs. The data show the ratio to the expression level of the ApEF1a gene. No data are presented for some growth stages at which we could not detect any PCR amplification because the target transcript levels were low. Scale bars: 50μm. (TIF) [file pone.0149067.s003.tif]

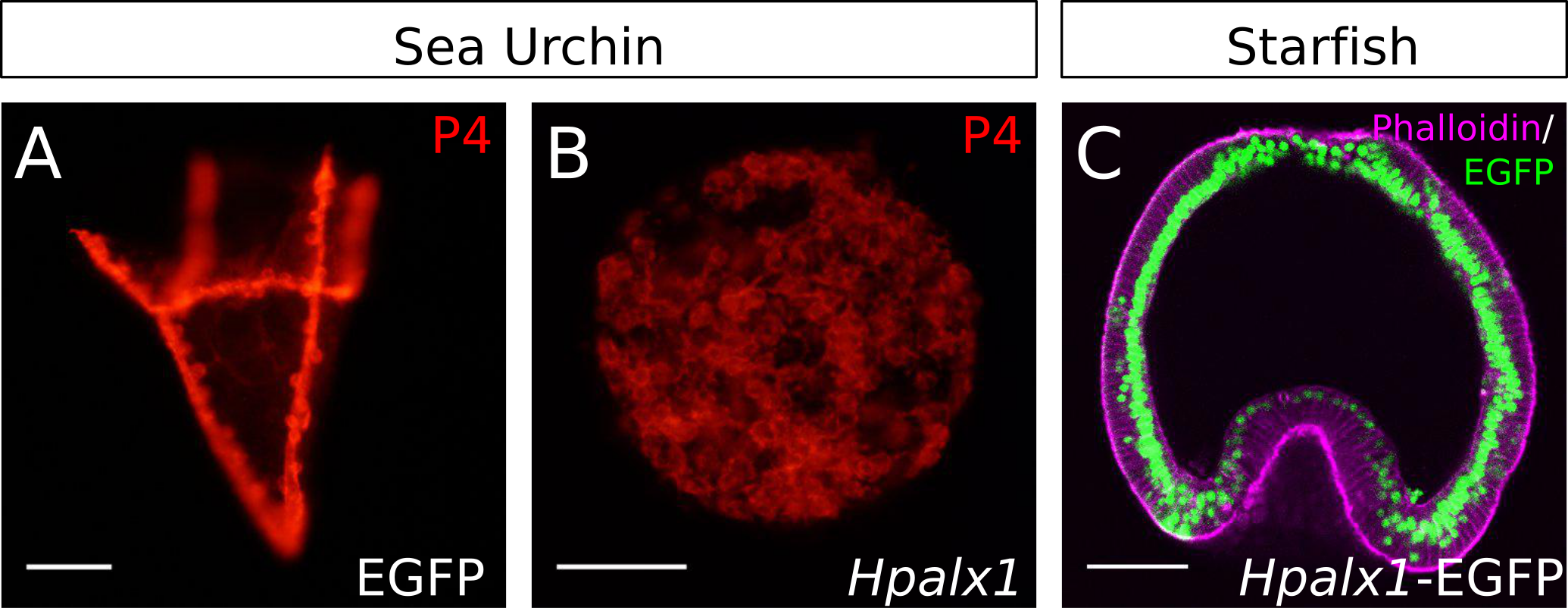

Supplement: S2 Fig — (A, B) Skeletogenic cells were stained by P4 antibody that recognizes Msp130 protein in 48 h postfertilization (hpf) of sea urchin larvae in which EGFP or Hpalx1 was overexpressed. (A) When mRNA encoding EGFP was injected, the embryos developed into normal plutei, with aligned skeletogenic cells. (B) When mRNA encoding HpAlx1 was injected, the embryos were abnormal, in that excessive numbers of skeletogenic cells were produced. (C) A fusion mRNA encoding both HpAlx1 and EGFP was injected into starfish eggs (the solution was 0.5 mg/ml in mRNA). EGFP signals were observed in almost all nuclei of a 15 hpf larva upon confocal microscopy. Magenta indicates staining for phalloidin. Scale bars: 50 μm. (TIF) [file pone.0149067.s004.tif]

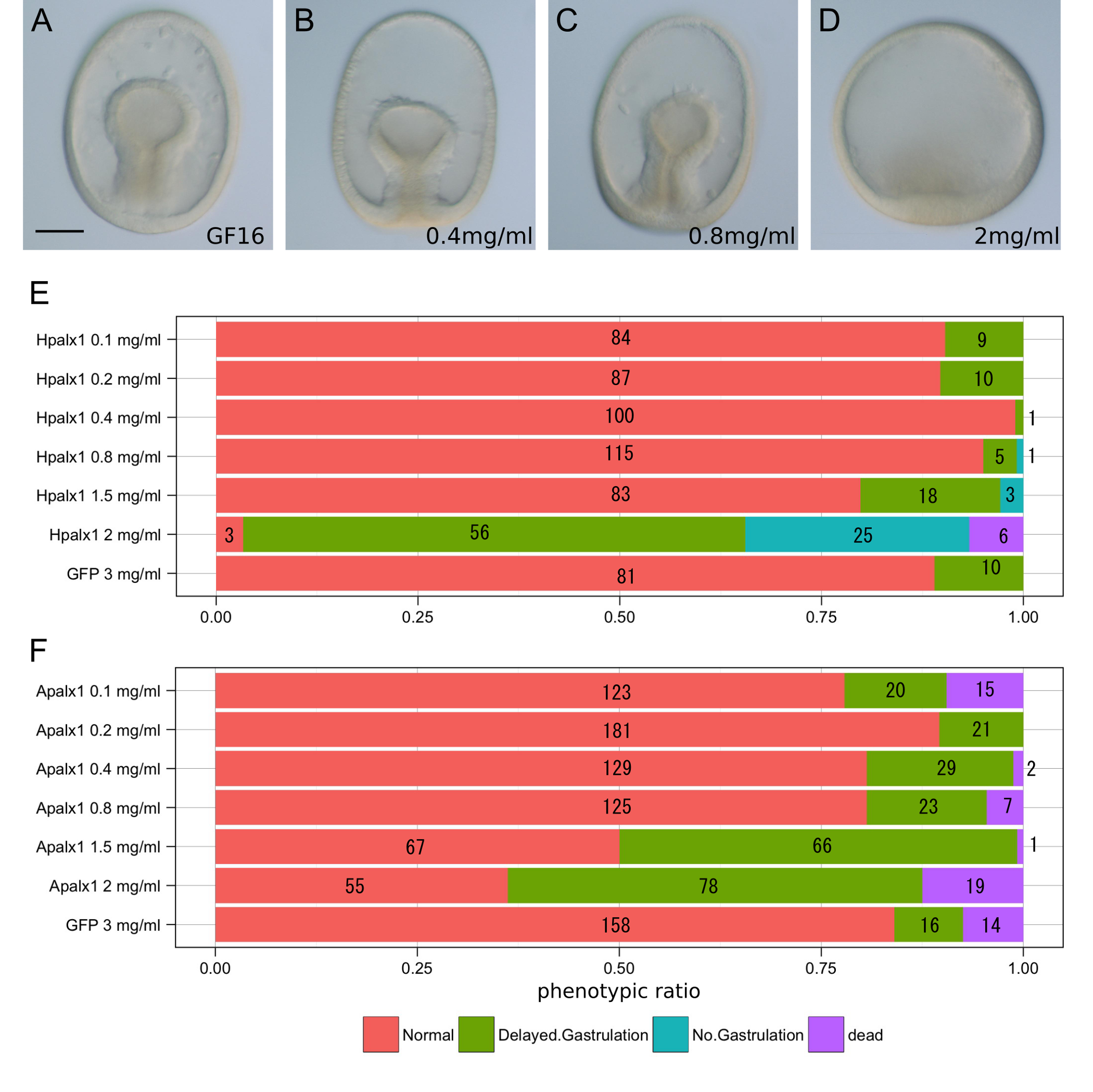

Supplement: S3 Fig — (A–D) mRNAs were injected at various concentrations; 24 hpf (hours postfertilization) larvae are shown. Scale bar: 50 μm. (A) When a concentrated (3 mg/ml) EGFP mRNA solution was injected, larvae developed normally to the gastrula stage. (B, C) When a lower concentration (0.1–0.8 mg/ml) of Hpalx1 mRNA was used, the injected larvae developed normally like the controls. (D) When a concentrated (2 mg/ml) Hpalx1 mRNA solution was injected, many larvae exhibited disturbances in gastrulation. The embryos shown did not commence gastrulation. (E, F) The graphs indicate the numbers of larvae exhibiting certain phenotypes when (E) sea urchin Hpalx1 mRNA or (F) starfish Apalx1 mRNA solutions of various concentrations were injected. All phenotypes were assessed at 48 hpf. (TIF) [file pone.0149067.s005.tif]

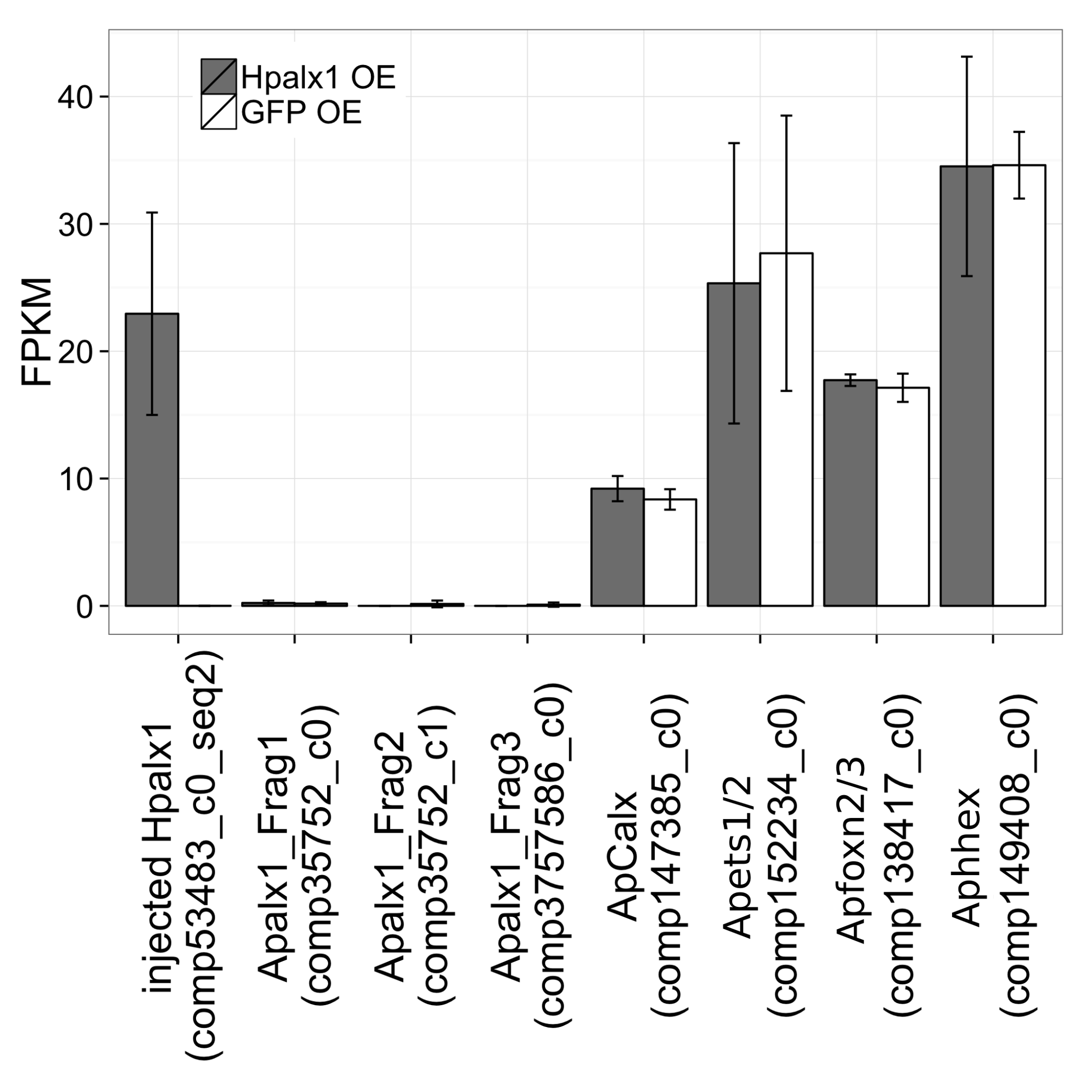

Supplement: S4 Fig — FPKM values calculated from read mapping results were shown (mean ± S.D.). In the Hpalx1-overexpressed sample, injected mRNA was detected and showed similar expression level to ets1/2, hhex and foxn2/3. While three Apalx1 fragments exhibited quite low FPKM values, ApCalx showed higher expression level. (TIF) [file pone.0149067.s006.tif]

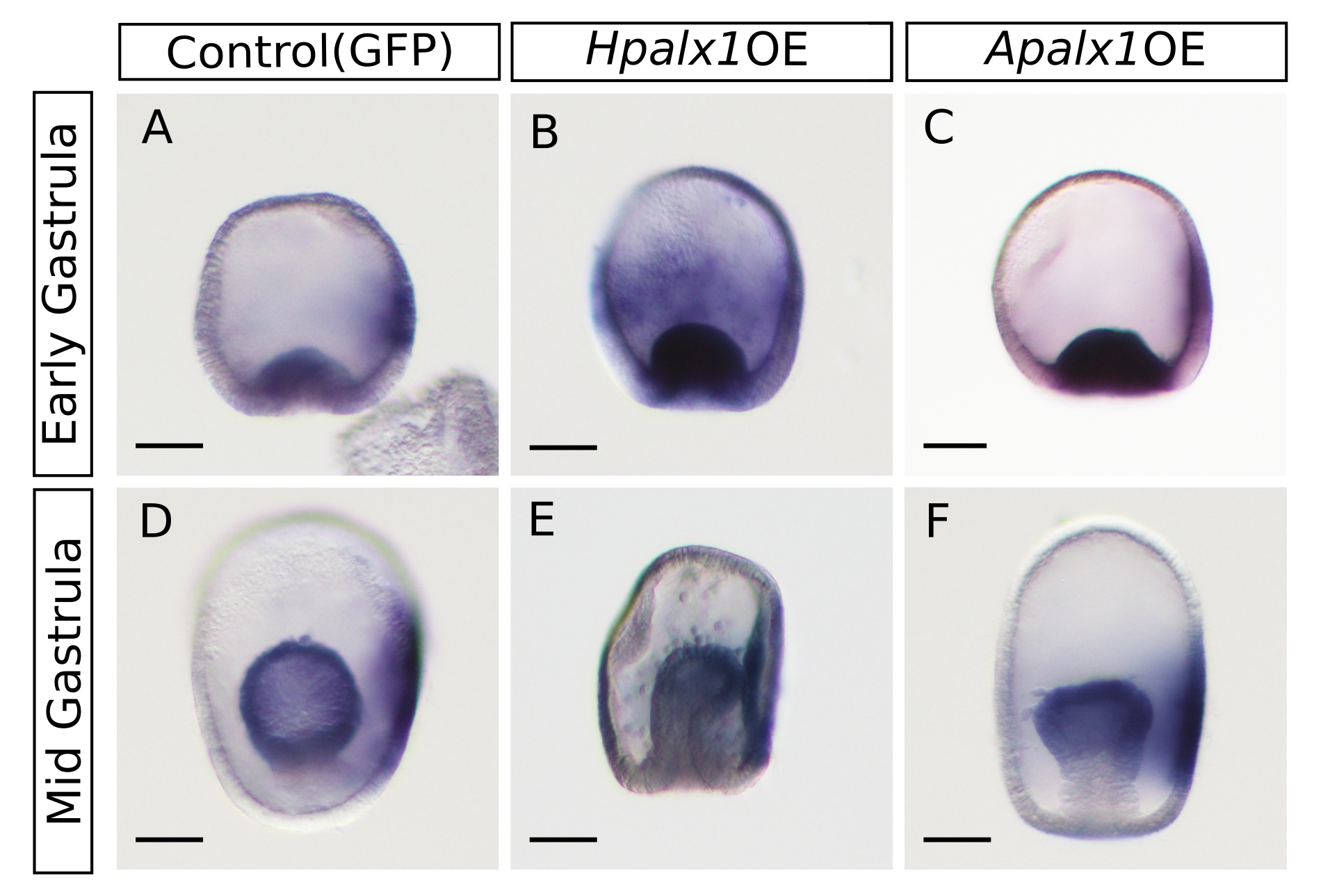

Supplement: S5 Fig — Solutions 1 mg/ml of EGFP (A, D), 0.5 mg/ml in mRNA for Hpalx1 (B, E) or 1 mg/ml in mRNA for Apalx1 (C, F) were injected into starfish eggs, which were next reared for 15 h (to the early gastrula stage) or 24 h (to the mid-gastrula stage). (A) The expression was observed in the invaginating archenteron and putative oral ectoderm in control early gastrula. (B) Sea urchin alx1 over expressed embryos showed basically same expression pattern as control embryo. The putative oral ectoderm is far side in this picture. (C) Starfish alx1-overexpressed embryos showed the same expression pattern as control. (D) In mid gastrula stage, the expression was observed in mesoderm tissue at the tip of archenteron as well as putative oral ectoderm. (E, F) Almost identical expression patterns were observed in both sea urchin and starfish alx1 overexpressed gastrulae. (TIF) [file pone.0149067.s007.tif]

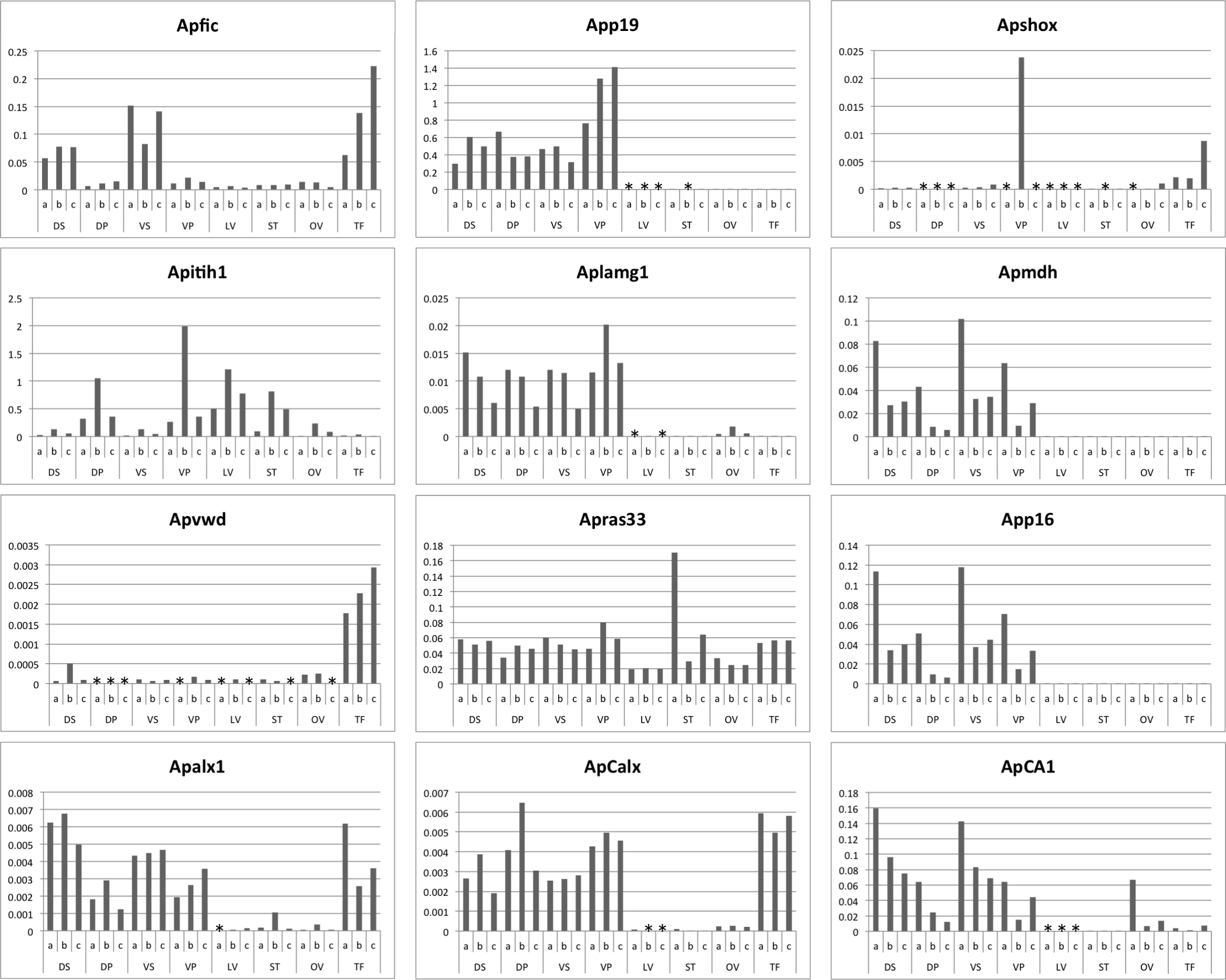

Supplement: S6 Fig — Expression levels relative to that of the EF1a gene were measured via real-time PCR. Results from three individual females are shown. Four skeletal tissues were examined: the dorsal (aboral) plates (DPs), the dorsal spines (DSs), the ventral (oral) plates (VPs), and the ventral spines (VSs). We also tested four nonskeletal tissues: the liver (LV), stomach (ST), ovaries (OV), and tube feet (TF). (TIF) [file pone.0149067.s008.tif]
