# Supplementary material for: Experimental Approach Reveals the Role of alx1 in the Evolution of the Echinoderm Larval Skeleton
Source: PLoS One. 2016 Feb 11;11(2):e0149067. doi: 10.1371/journal.pone.0149067 (PMC4750990; doi:10.1371/journal.pone.0149067)
Supplement: S1 Table — (DOCX) [file pone.0149067.s009.docx]

S1 Table. Results of the *de novo* transcriptome assemblies

| Species | *A. pectinifera* (injected) | *H. leucospilota* | *M. rotundus* |
| --- | --- | --- | --- |
| Number of putative genes (Trinity Sub-components) | 107,286 | 138,030 | 123,355 |
| Number of contigs (Trinity Seqs) | 206,700 | 214,145 | 163,991 |
| N50 of contigs | 1,954 | 1,454 | 815 |
| Average length of contigs | 1026.94 | 819.77 | 612.31 |
