# Supplementary material for: Experimental Approach Reveals the Role of alx1 in the Evolution of the Echinoderm Larval Skeleton
Source: PLoS One. 2016 Feb 11;11(2):e0149067. doi: 10.1371/journal.pone.0149067 (PMC4750990; doi:10.1371/journal.pone.0149067)
Supplement: S2 Table — (DOCX) [file pone.0149067.s010.docx]

S2 Table. Alx proteins used in the phylogenetic analysis

| Echinoderm |  |  |  |  |
| --- | --- | --- | --- | --- |
| Class | Species | Name | Source | ID* |
| Crinoidae | *Metacrinus rotundus* | MrAlx1 | RNAseq | LC005505^†^ |
| (sea lilies) |  | MrCalx | RNAseq | LC005506^†^ |
| Asteroidea | *Asterina (Patiria) pectinifera* | ApAlx1 | cDNA | LC005499^†^ |
| (starfish) |  | ApCalx | cDNA | LC005500^†^ |
|  | *Asterina (Patiria) miniata* | AmAlx1 | Genbank | AFS65546 |
| Ohiuroidea | *Amphipholis kochii* | AkAlx1 | cDNA | LC005501^†^ |
| (brittle star) |  | AkCalx | cDNA | LC005502^†^ |
| Holothuroidea | *Holothuria leucospilota* | HlAlx1 | RNAseq | LC005503^†^ |
| (sea cucumber) |  | HlCalx | RNAseq | LC005504^†^ |
| Echinoidea | *Strongylocentrotus purpuratus* | SpAlx1 | Spbase | SPU_025302 |
| (sea urchin) |  | SpAlx4 | Spbase | SPU_022816 |
|  | *Hemicentrotus pulcherrimus* | HpAlx1 | cDNA | LC005497^†^ |
|  |  | HpCalx | cDNA | LC005498^†^ |

S2 Table continued.

| Outgroups |  |  |  |  |
| --- | --- | --- | --- | --- |
| phylum | Species | Name | Source | ID* |
| Hemichordata | *Saccoglossus kowalevskii* | SkAlx | Genbank | XP_002735897 |
| Chordata | *Branchiostome froridae* | BfAlx1 | Genbank | XP_002590991 |
|  | (Amphioxus) | BfAlx2 | Genbank | XP_002590993 |
|  | *Danio rerio* (zebrafish) | DrAlx1 | Uniprot | CAK04167 |
|  |  | DrAlx4 | Genbank | XP_001340966 |
|  | *Homo sapiens* (Human) | HsAlx1 | Uniprot | Q15699 |
|  |  | HsAlx3 | Uniprot | O95076 |
|  |  | HsAlx4 | Uniprot | Q9H161 |
| * Accessions for Spbase (www.spbase.org), GenBank or Uniprot  † Genes deposited by this work | | | | |
