# Supplementary material for: Experimental Approach Reveals the Role of alx1 in the Evolution of the Echinoderm Larval Skeleton
Source: PLoS One. 2016 Feb 11;11(2):e0149067. doi: 10.1371/journal.pone.0149067 (PMC4750990; doi:10.1371/journal.pone.0149067)
Supplement: S3 Table — (DOCX) [file pone.0149067.s011.docx]

S3 Table. PCR primers for isolation of *Alx* and other genes.

| Target | Forward primer (5’-3’) | Reverse primer (5’-3’) | usage |
| --- | --- | --- | --- |
| *Hpalx1* | ATGTTGTTTTACCCCTCAAT | GGATGATGAACGGTTACTCG |  |
| *HpCalx* | ATGACAAACGAGAGTTTGAAGTTGTTGCA | TTAAGAGTAAGCGCCTATCATTCCCATGGC |  |
| *Apalx1* | ATGCTCAATCTGGTCGGCCGGT | GGAGTATCCGTTCATCATTCC |  |
| *ApCalx* | CGGCACGAGGTTGCTCGCTTCAATTATACT | CAATCAGTATACGATGAGCCACTGATGGGC |  |
| *App19* | GACAGTGAAGTTATTGGTTTTAAG | ACTACATCTACTGAATTTTAAGGAAAG |  |
| *App16* | CACCAAAGTTTTACACATCTAACTCG | TCACTAGAAAGACCAATAGTCAAACCT |  |
| *Apdri* | GAGGTCCTCATTAGAAAGAGAGAGAA | GGTCTATGGGAGTCGATTTATAGGT |  |
| *Akalx1* | GCAAGAGTACAGGTATGGTTTCAG | - | 3’RACE |
|  | ACAGGTATGGTTTCAGAACCG | - | 3’RACE Nested |
| *AkCalx* | AGAGAACAGCTGGCGTTGAGGTGTG | - | 3’RACE |
|  | CCGATGGCACCAAGACCAGAAAGTT | - | 3’RACE Nested |
| *Hlalx1* | GGCTCCGAGAGGAGAATATGGACAGGT | - | 3’RACE |
|  | CTTGTATGGTTCCACAGGGAGGCCTTC | - | 3’RACE Nested |
| *Bsimalx* | ACTGTCGTGAACAGTTAGCGTTGCGTTG | - | 3’RACE |
|  | GGACTTAGACACAAAAAC | - | 3’RACE Nested |
