# Supplementary material for: Experimental Approach Reveals the Role of alx1 in the Evolution of the Echinoderm Larval Skeleton
Source: PLoS One. 2016 Feb 11;11(2):e0149067. doi: 10.1371/journal.pone.0149067 (PMC4750990; doi:10.1371/journal.pone.0149067)
Supplement: S4 Table — (DOCX) [file pone.0149067.s012.docx]

**S4 Table. Primer sets for real-time PCR.**

| Target Gene | Name | Forward_Primer (5'-3') | Reverse_Primer (5'-3') | Fitted Control |
| --- | --- | --- | --- | --- |
| *ApEF1a* | ApEF1a_1 | GCCTCTTCGTCTACCTCTGC | CGACACCTGGCTTGATGATA | - |
| *ApEF1a* | ApEF1a_2 | ACGTAGGCTTCAACGTCAAG | ATTTGCCCGGGATGGTTCAG | - |
| *ApEF1a* | ApEF1a_3 | TGGCGTTCGTTCCCATTTC | GTTCAATTTCCCAACCCTTGTAC | - |
| *ApEF1a* | APEF1a_4 | ACAGCGGCCTTCAGATAAGC | ACGACACCTGGCTTGATGATAC | - |
| *Apalx1* | Apalx1 | ACTCACAGCATGCAGATTATGG | CATAGGCGCGGGATTCATG | ApEF1a_3 |
| *ApCA1* | ApCA1 | CATGGACGGTGTTCAAAATG | AATCGGGATGTCAGGAGATG | ApEF1a_1 |
| *ApCalx* | ApCalx | GGGGGACTACTCGTGCTATC | GCTTGTCCGCTCGATGTC | ApEF1a_2 |
| *Apdri* | Apdri | ATACCTCACCCATCAGCCATTC | ATTGGGTGTTTGGCCAATCG | ApEF1a_2 |
| *Aperg* | Aperg | CGCTTGCAAATCCAGAAAACC | CTCCCACGTGATGCAGTTG | ApEF1a_3 |
| *Apets1/2* | Apets | ATGATGTTTGACCCCAACGC | CACCTGCTGGTAGCCGTTAG | ApEF1a_2 |
| *Apfic* | Apfic | ACGAGTACCAGTCTGGCTTTG | TCAACTCGTAGCTCCCATGTTC | ApEF1a_4 |
| *Apfoxb* | Apfoxb | CCAGTCCAAACACGGTTTCATC | ATCCGGCGAGATGATGTTCTC | ApEF1a_2 |
| *Apfoxn2/3* | Apfoxn23 | TCGCATCCCTACCAGTATCATC | TGCCATTACTCCAGCCAATG | ApEF1a_2 |
| *Apfoxo* | Apfoxo | CGACGGCAACCTAGATTTCAAC | GCATTCCAAGCTGCGTTCTG | ApEF1a_1 |
| *Aphhex* | Aphhex | ACCCACACGGACTAATCGATAG | TGAAGGGGTTCCAGAGAAATGG | ApEF1a_2 |
| *Apitih1* | Apitih1 | CGCTCCACAAAGTGATCCAG | GCCATTTTCAGAACGCAGTC | ApEF1a_2 |
| *Aplamg1* | Aplamg1 | CCTTGTCAGAATGATGGCAGTTG | TGTTGGTGAAGGAGACCACATC | ApEF1a_3 |
| *Apmdh1* | Apmdh1 | GAGCTCTGAACAGGTGTTGAAG | ATGCGATGACGGTTGTTCTG | ApEF1a_3 |
| *App16* | App16 | CACATCTAACTCGAGCGTCTTC | ATGTGGAAGAACCGAGGATG | ApEF1a_2 |
| *App19* | App19 | TTCACAAGCACCGGAACAAC | TGCGGCTGTAGACTGATTACTG | ApEF1a_1 |
| *Aprab33* | Aprab33 | TGGACGTTGCGGTAGTAGTG | ACGATAGGGGTGGATTTCAG | ApEF1a_1 |
| *Apshox* | Apshox | ATTTGCTTGGGCTCAGTGAC | CCACAGCAGTTGAGCAAATC | ApEF1a_2 |
| *Aptbr1* | Aptbr | AAGTTCCACGAGCATCGAAC | TGGGATCTGCTATGACCATGTC | ApEF1a_3 |
| *Aptel* | Aptel | ATAACCCTGGAAAGGCACCTG | TTTGGAGCTTGGCATCTTGC | ApEF1a_3 |
| *Aptgif* | Aptgif | ACGTTTTCCCTTCCTTCGTC | ATCTGATGCTCCACTGTCAGAG | ApEF1a_1 |
| *Apvegfr* | Apvegfr | AACACCGAGGCTAAGGAAAGAC | GGCTGGCGTTCTCAATTTCG | ApEF1a_2 |
| *Apvwd* | Apvwd | GATTATCCGATCCTCCATGC | GGATGACTGGATGAAGCAAG | ApEF1a_1 |
